# PolyMyalgia Rheumatica treatment with Methotrexate in Optimal Dose in an Early disease phase (PMR MODE): study protocol for a multicenter double-blind placebo controlled trial

**DOI:** 10.1186/s13063-022-06263-3

**Published:** 2022-04-15

**Authors:** Diane E. Marsman, Thomas E. Bolhuis, Nathan den Broeder, Alfons A. den Broeder, Aatke van der Maas

**Affiliations:** 1 Department of Rheumatology, Sint Maartenskliniek, Nijmegen, The Netherlands; 2 Radboud Institute for Health Sciences, Radboud University Medical Center, Nijmegen, The Netherlands; 3 Department of Rheumatic Diseases, Radboud University Medical Centre, Nijmegen, The Netherlands

**Keywords:** Polymyalgia rheumatica, Early disease, Methotrexate, Glucocorticoid sparing, Randomized controlled trial

## Abstract

**Background:**

Polymyalgia rheumatica (PMR) is an inflammatory rheumatic disease affecting people older than 50, resulting in pain and stiffness of the neck, shoulder, and pelvic girdle. To date, glucocorticoids (GC) remain the cornerstone of treatment, but these have several drawbacks. Firstly, a large proportion of patients do not achieve GC-free remission within either the first (over 70%) or second year of treatment (over 50%). Secondly, GC-related adverse events (AE) occur in up to 65% of patients and can be severe.

The current EULAR/ACR guidelines for PMR recommend early introduction of methotrexate (MTX) as a GC sparing agent in patients at risk for worse prognosis. However, earlier trials of low to medium quality only studied MTX dosages of 7.5–10 mg/week with no to modest effect. These doses may be suboptimal as MTX is recommended in higher doses (25 mg/week) for other inflammatory rheumatic diseases. The exact role, timing, and dose of MTX in PMR remain unclear, and therefore, our objective is to study the efficacy of MTX 25 mg/week in recently diagnosed PMR patients.

**Methods:**

We set up a double-blind, randomized, placebo-controlled superiority trial (PMR MODE) to assess the efficacy of MTX 25 mg/week versus placebo in a 1:1 ratio in 100 recently diagnosed PMR patients according to the 2012 EULAR/ACR criteria. All patients will receive prednisolone 15 mg/day, tapered to 0 mg over the course of 24 weeks. In case of primary non-response or disease relapse, prednisolone dose will be temporarily increased. Assessments will take place at baseline, 4, 12, 24, 32, and 52 weeks. The primary outcome is the difference in proportion of patients in GC-free remission at week 52.

**Discussion:**

No relapsing PMR patients were chosen, since the possible benefits of MTX may not outweigh the risks at low doses and effect modification may occur. Accelerated tapering was chosen in order to more easily identify a GC-sparing effect if one exists. A composite endpoint of GC-free remission was chosen as a clinically relevant endpoint for both patients and rheumatologist and may reduce second order (treatment) effects.

**Trial registration:**

Dutch Trial Registration, NL8366. Registered on 10 February 2020

## Administrative information

Note: the numbers in curly brackets in this protocol refer to SPIRIT checklist item numbers. The order of the items has been modified to group similar items.

**Table:** 

Title {1}	The efficacy of Methotrexate for glucocorticoid dose reduction in recently diagnosed polymyalgia rheumatica patients: a double-blind randomized placebo controlled multicenter clinical trial.
Trial registration {2a and 2b}.	Dutch trial registration, NL8366 Registered on 2020-02-10 (CMO Regio Arnhem-Nijmegen NL69979.091.19, date 2020-01-23).
Protocol version {1}	20-07-2021 version 2.1
Funding {4}	This is an investigator driven trial and (partially) funded by ReumaNederland (funding number 18-2-401)
Author details {5a}	Department of Rheumatology, Sint Maartenskliniek, Hengstdal 3, 6574 NA Nijmegen, The Netherlands Radboud Institute for Health Sciences, Radboud university medical center, Nijmegen, The Netherlands. Department of Rheumatic Diseases, Radboud Institute of Health Sciences, Radboud University Medical Centre, Nijmegen, The Netherlands
Name and contact information for the trial sponsor {5b}	Dr. Aatke van der Maas. Sint Maartenskliniek, Department of Rheumatology. PO box 9011, 6500 GM Nijmegen, The Netherlands A.vandermaas@maartenskliniek.nl fax number +31 24 3659743 telephone number. +31 24 3659985
Role of sponsor {5c}	The study funder had no role in the study design, collection, management and analysis. The study funder will not have a role in the interpretation of data; writing of the report; and the decision to submit the report for publication, nor will they ultimate have authority over any of these activities.

## Introduction

### Background and rationale {6a}

Polymyalgia rheumatica (PMR) is an inflammatory rheumatic disease affecting mostly people older than 50 years [1]. Patients generally present with subacute onset pain and stiffness of the neck, bilateral shoulder, and pelvic girdle, and elevated acute phase reactants [1–3]. Additionally, 40–50% of patients may report constitutional symptoms. PMR is closely related to giant cell arteritis (GCA), a large blood vessel vasculitis (LVV) occurring in elderly people [1]. The cause of PMR remains unknown and there is no golden standard for the diagnosis of PMR [1, 4]. The duration of the disease can be up to 2–3 years, and during the first year, the chance of relapse can range up to 20–55% [1, 3, 5, 6]. Untreated PMR leads to a significant reduction in quality of life (QOL) [5].

To date, glucocorticoids (GC) remain the cornerstone of treatment of PMR [7]. Several different tapering regimens exist, but there is no data on which regimen is optimal, as these have not been adequately investigated [7]. Firstly, there may be a long treatment duration due to lack of a true disease-modifying effect; GC-free remission was achieved in only 27% in a PMR primary care cohort within the first year of treatment [8] and in only 33–50% of a hospital care cohort of PMR patients after 2 years of treatment [5, 9]. Secondly, GC-related adverse events (AE) are frequent and have been reported in up to 65%, dependent on GC dosage [5, 10–12]. Patients using GCs longer than 2 years are more likely to develop weight gain, osteoporosis, fractures, metabolic and cardiovascular side effects as well as infections [5, 10, 11, 13]. Thirdly, studies report relapse percentages of up to 50% [3, 5, 6]. These relapses can disrupt tapering, increasing treatment duration, GC dose, and consequent GC related AE. This emphasizes the need for GC sparing agents to enhance GC efficacy, shorten treatment duration, and reduce GC-related side effects.

The efficacy of several disease-modifying antirheumatic drugs (DMARD) as GC sparing agents has been studied but the exact role in PMR remains unclear. To date, there is no proven efficacy or data is insufficient on azathioprine, cyclophosphamide, cyclosporin, and dapsone [14]. For leflunomide, there are two case series that showed some promise of efficacy in PMR patients [15, 16]. Of biological DMARD treatment, tocilizumab shows some promise, although it is associated with high costs and due to its effect on C-reactive protein (CRP) disease monitoring might be somewhat impaired [17, 18]. Furthermore, a recent small randomized controlled trial (RCT) showed some efficacy of rituximab, although these results still need to be confirmed in a larger and longer RCT [19].

The most evidence—three small RCTs—for a GC-sparing treatment exists for methotrexate (MTX), although the quality of evidence is still low [7, 14, 20–22]. Firstly, the examined doses varied between 7.5 and 10 mg once weekly, and no studies are available on MTX 25–30 mg, which is used for other inflammatory rheumatic diseases and is clearly more effective [23, 24]. Furthermore, the studies were small, limited by high drop-out rates, and in some studies open label MTX use. The results of these studies are also conflicting, as only one study with 10 mg showed some efficacy by reduced relapse rate and GC-dose [22] and the other two studies did not show an effect [20, 21].

However, based on this sparse evidence, the current European League Against Rheumatism / American College of Rheumatology (EULAR/ACR) recommendations for the management of PMR advise an early introduction of MTX in patients with worse prognosis such as in patients prone to relapse or prolonged GC-therapy, as well as in patients where GC-related AE are more likely to occur [4, 7, 25]. In clinical practice, however, MTX is infrequently used, as reported by a national database study of Albrecht et al., who found that only 19% of patients with PMR received concomitant MTX [10]. This curbed use may reflect the uncertainty of the exact role of MTX in PMR, due to the limited and conflicting evidence. Therefore, further research regarding the use of MTX is high on the research agenda of the 2015 EULAR/ACR guideline for the management of PMR [7].

In conclusion, evidence on GC-sparing treatment for PMR patients remains scarce and MTX seems to be a reasonable candidate to study. We therefore set up a double-blind placebo controlled trial of MTX 25 mg once weekly in early PMR patients to assess whether higher dosed MTX, started early, is effective in achieving GC-free remission and reducing cumulative GC dose in PMR.

### Objectives {7}

The main objective of this study is to determine whether add-on MTX 25 mg/week compared to placebo is more efficacious in increasing the proportion of recently diagnosed PMR patients in GC-free remission after 52 weeks of treatment. Secondary outcomes are other outcomes related to disease activity, physical function, health-related quality of life, and GC- and MTX-related AE, at different time points in the study.

### Trial design {8}

This is an investigator driven, multicenter, double-blind, randomized, placebo-controlled superiority trial.

## Methods: participants, interventions, and outcomes

### Study setting {9}

The study is a multicenter study that will be conducted in the Sint Maartenskliniek (at the outpatient rheumatology clinics located in Nijmegen, Woerden, Boxmeer, Geldrop) and Gelre ziekenhuizen (at the outpatient rheumatology clinics located in Apeldoorn and Zutphen) in The Netherlands. Patients will be recruited over the course of 24 months, but as timely recruitment may be challenging, we are aiming to include other centers.

### Eligibility criteria {10}

#### Inclusion criteria

For this study, we will include patients with recently (within the last 12 weeks) diagnosed PMR according to the 2012 EULAR/ACR preliminary classification criteria.

#### Exclusion criteria

Our main exclusion criteria are GC exposure for > 8 weeks; GC treatment with > 30 mg/day, and exposure to other systemic immunosuppressant treatment other than GC 3 months prior to inclusion in the study. We chose for a short GC exposure duration since we want to know the additive effect of MTX given early in the disease course, and this shorter period also increases homogeneity of patients. Additionally, we think that a GC need > 30 mg/day requires considering different diagnoses. The decision to exclude patients treated with other DMARDs 3 months prior to inclusion is because we want to be certain that any GC-sparing effect we see in our study is only due to MTX and not due to carry over effect from previous treatments. Also, to ensure adequate assessments, we will exclude patients who do not show willingness to follow study protocol; with an inadequate ability to speak, read, or write Dutch; and with active concomitant GCA or other rheumatic diseases such as RA, spondyloarthropathies, connective tissue diseases, drug-induced myopathies, neuropathies, or other conditions that might interfere with pain or movement evaluation of PMR, or interfere with treatment choices with respect to GC and DMARDS.

### Who will take informed consent? {26a}

The research physician will acquire informed consent in duplicate and one copy will be given to the patient. Patients may withdraw their informed consent at any time.

### Additional consent provisions for collection and use of data {26b}

Separate informed consent is taken for collecting additional (biobanking) samples when blood is taken, further elaborated upon in SPIRIT header {33}. Separate approval will be sought for PMR-related research regarding these samples.

### Interventions

#### Explanation for the choice of comparators {6b}

MTX has been noted as a “potential steroid saving” drug in the treatment of PMR in the EULAR/ACR guidelines [7]. Furthermore, MTX is the first choice of DMARD in RA, and one of the most prescribed drugs in comparison to other DMARDS [23, 24]. Three earlier studies have compared the effect of MTX with placebo with an accelerated prednisone scheme, but the MTX dose used may have been suboptimal [20, 26]. In this study, we chose oral MTX over subcutaneous MTX for several reasons. Firstly, in discussion with patients they indicated a preference for oral MTX as there was a reluctance against MTX injections. Secondly, the MTX and placebo capsules are less expensive compared to subcutaneous injections. Thirdly, efficaciousness of oral MTX may be non-inferior compared with subcutaneous MTX with regard to bioavailability and efficacy [27, 28]. Furthermore, oral MTX will be spread out in two doses over the course of a day in increase bioavailability and decrease potential AE. In this study, we chose for an accelerated trial and error GC-tapering protocol (of 24 weeks)—which is approximately twice as fast as usual care— for several reasons. Firstly, optimal GC-tapering in PMR is unknown as this is based on limited evidence of low to medium quality [7]. Secondly, minimizing GC treatment may minimize AE [13]. Thirdly, and potentially most importantly, minimizing GC treatment will make it easier to identify a possible effect of MTX on GC use and GC free remission.

#### Intervention description {11a}

After inclusion, patients will randomly be allocated into one of two arms with a 1:1 ratio. Patients allocated to the treatment arm will start with oral MTX 15 mg per week, which will be increased to 25 mg per week after 4 weeks for the remainder of the study period if no clinically relevant MTX-related side effects occur (Table 1). Patients assigned to the placebo arm will receive an identical amount of indistinguishable placebo capsules, containing MTX 0 mg, once weekly. MTX will be dosed in capsules of 5 mg to allow easier dose adjustment without the risk of un-blinding treatment. Also, it will be easier to split the dose over the course of the day to increase bioavailability. After the 52 week study period, study medication will be stopped.

**Table 1:** Treatment set-up for initial responders without relapse

	Time in weeks	0	4	8	12	16	20	24	28	32	36	40	44	48	52
**MTX group**	Prednisolone (mg/day)	15	12.5	10	7.5	5	2.5	0	0	0	0	0	0	0	0
	MTX (mg/week)	15	25	25	25	25	25	25	25	25	25	25	25	25	25
	Folic acid (mg/week)	10	10	10	10	10	10	10	10	10	10	10	10	10	10
**Placebo group**	Prednisolone (mg/day)	15	12.5	10	7.5	5	2.5	0	0	0	0	0	0	0	0
	MTX (mg/week)	0	0	0	0	0	0	0	0	0	0	0	0	0	0
	Folic acid (mg/week)	10	10	10	10	10	10	10	10	10	10	10	10	10	10

**Fig. 1:**
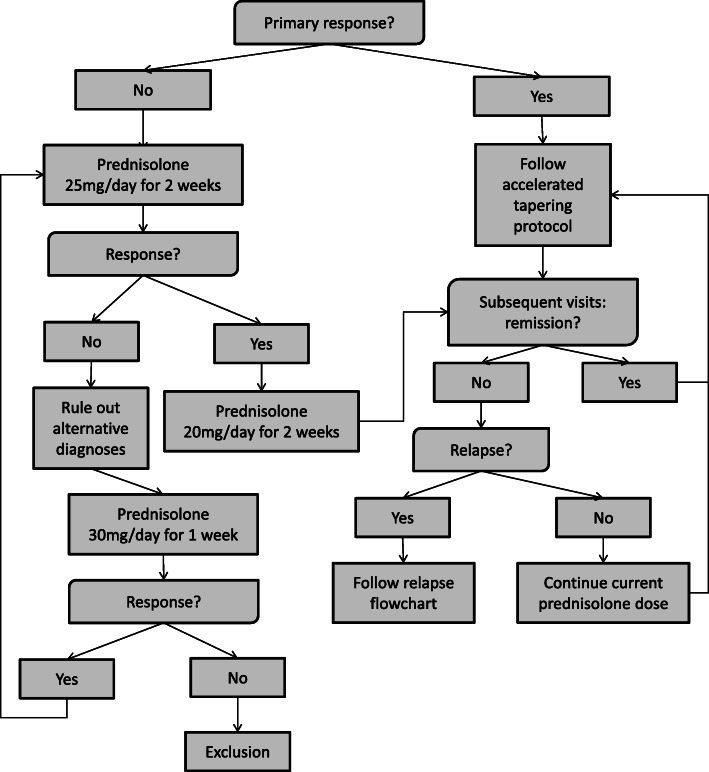
Treatment protocol flowchart {11b}

**Fig. 2:**
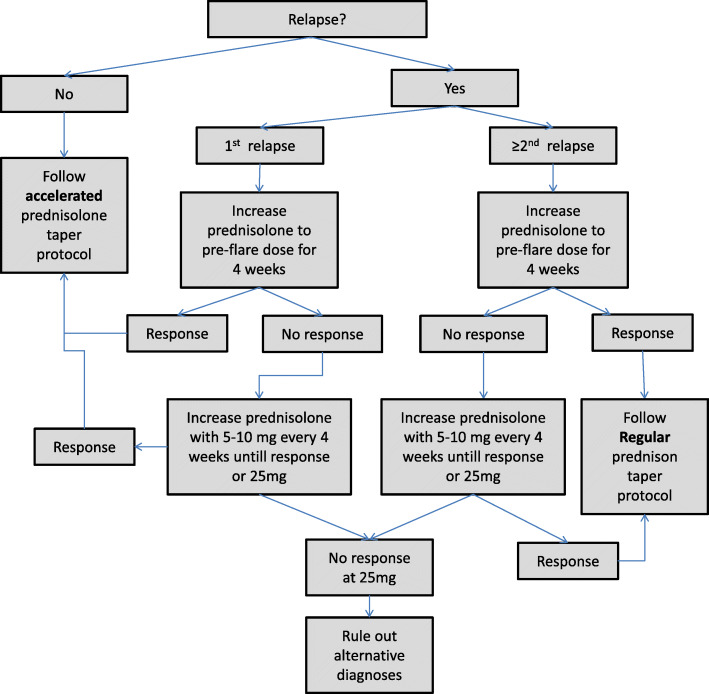
Relapse flowchart {11b}

Patients in this study will start with a prednisolone dose 15 mg once daily, followed by an accelerated disease activity guided tapering scheme over the course of 24 weeks, with a dose reduction of 2.5 mg every 4 weeks (Table 1). Prednisolone will only be tapered after acquiring an adequate initial response and in the absence of a disease relapse. If primary non-response occurs during the first 4 weeks, the prednisolone dose will be increased to 25 mg/day for 2 weeks, followed by 20 mg/day for 2 weeks and subsequently 15 mg/day followed by the study tapering protocol (Fig. 1). When no primary response is obtained after treatment with 25 mg/day, alternative diagnoses such as GCA will be ruled out. If a patient does not respond after a maximum of 4 weeks, prednisolone can be raised further to 30 mg/day for 1 week. If a patient does not respond in this time, they will be excluded from the study and replaced by a new patient, because we think an alternative diagnosis is more likely. If a relapse occurs after initial primary response, prednisolone dose will be increased to the pre-relapse dose, followed by tapering in the case of response, or further raising of the dose in the case of non-response. If this is the first relapse, tapering will occur according to the study tapering protocol. If a second relapse occurs tapering will occur according to usual care. See Fig. 2 for a detailed description of the relapse protocol. Additionally, all patients will receive folic acid 5 mg twice weekly, to reduce potential MTX related side effects [29]. Osteoporosis prophylaxis for prevention of GC-related osteoporosis will be given if indicated [30].

#### Criteria for discontinuing or modifying allocated interventions {11b}

Since MTX can lead to hepatotoxicity or blood count abnormalities, monitoring of hepatoxicity will be done by ALAT serum levels and blood count by hemoglobin level, leukocyte count, and platelet count according to the Dutch Rheumatologist Association guidelines for methotrexate treatment [31]. If ALAT serum level is more than 3 times the upper bound of the normal values, leukocyte count is < 3.0 × 10^9^/L, or platelet count is < 100 × 10^12^/L; testing will be repeated within 7 days to determine whether these values improve, stabilize, or worsen. If laboratory abnormalities are still clinically relevantly elevated, as judged by treating physician, then MTX (or placebo) dose may be skipped for that week. Furthermore, laboratory values will be monitored the week thereafter and MTX (or placebo) dose can either be adjusted to a minimum of 10 mg/week or continued. If clinically relevant lab abnormalities persist, MTX or placebo will be stopped, and patients will remain in the study. Treatment of other AE is left to the discretion of the treating physician.

#### Strategies to improve adherence to interventions {11c}

If patients were not able to adhere to the treatment protocol, the reasons will be asked and noted by the treating physician. To improve MTX/placebo treatment adherence, in case the relatively frequent side-effect nausea occurs, ondansetron 4 mg 1–2 times daily can be prescribed. Other side effects will be treated as judged by the treating physician.

#### Relevant concomitant care permitted or prohibited during the trial {11d}

Patients are not allowed to take part in a competing clinical study while enrolled in this study. Patients can use paracetamol and non-steroidal anti-inflammatory drugs (NSAIDs) during the trial. Patients are encouraged to report any new medications, or medication changes, during the trial.

#### Provisions for post-trial care {30}

After completion of the trial, usual care will be provided. This might include open label MTX (MTX 2.5-mg tablets instead of 5-mg capsules in the Netherlands), or other treatment according to management guidelines.

### Outcomes {12}

The primary outcome of this study is the between-group difference in proportion of PMR patients in GC-free remission at week 52. This outcome captures both the state of disease activity and absence of GC-use. Combining these two in one composite measure is in our eyes a relevant and efficient way to measure the efficacy of MTX. Also, the 52-week endpoint is approximately half a year after the earliest possible end of GC treatment (at week 24). Thus, we think this reflects the more relevant longer term efficacy. Furthermore, we chose a point sufficiently far enough that patients with a relapse (that have prednisolone dose raised and again tapered) can still achieve GC-free remission, since PMR relapse occurs frequently and increased taper speed further increases chance of relapse [3]. Additionally, we chose GC-free remission instead of an outcome like GC cumulative dose, because in our opinion GC-free remission is more clinically relevant and pragmatic, both for patients and physicians, as it also enables calculating Numbers Needed to Treat. In PMR, there is no validated measure for disease activity but, since most evidence exists for the PMR-AS, we chose for a PMR-AS-based score to define remission [32–35]. The PMR-AS is discussed in more detail in the paragraph on assessments with SPIRIT header {18a}.

Secondary outcomes are the proportion of patients in GC-free remission at week 32; the time to GC-free remission and first relapse; the GC cumulative dose at week 32 and 52; the number of relapses or recurrences during follow-up at week 32 and 52; the proportion of patients that relapsed or had a recurrence during follow-up at week 32 and 52; the change in PMR-AS; the change in ESR, CRP, transition and PASS questions, VAS, EQ-5D, HAQ, and PROMIS-PF; the frequency and types of GC-related adverse events during the study as measured by the Glucocorticoid Toxicity Index (GTI); the frequency and types of GC- and MTX-related adverse events; and the proportion of patients with a MTX/placebo dose adjustment during follow-up at week 52. After approval of the amendment of the trial by the Medical Ethics Review Committee, the following secondary outcomes were added to the trial: the proportion of low-dose GC (≤ 5 mg daily) remission at weeks 32 and 52, and cost-utility (based on EQ-5D and direct healthcare costs). The choices of several of these secondary outcomes are based on the proposed inner core domains (systemic inflammation, physical function, pain, and stiffness) for PMR by the OMERACT working group [36]. Furthermore, we chose multiple patient-reported outcomes (PROs) to get a better insight in the quality of life and functioning of PMR patients.

We chose to use a modified version of the GTI developed by Miloslavsky et al. [37] because this enables a detailed and standardized assessment of GC-related AE. We chose to exclude standardized dual energy X-ray absorptiometry (DEXA) and cortisol assessment, as this is more pragmatical in implementation and reduces patient burden and costs. In PMR trials, there is much heterogeneity regarding outcome measures; in our opinion, using the inner core domain as proposed by the OMERACT working group and the GTI will increase the chance of homogeneity and comparability of this trial with other (future) trials of PMR.

### Participant timeline {13}

The pre-recruitment phase of the study is scheduled to take 12 months. The recruitment and inclusion phase is expected to take 24 months. Follow-up will take 12 months for each recruited patient. Data analysis, reporting, and submitting the written article of the study are scheduled to take 6 months. Therefore, total study time is approximately 52 months. We initially aimed to include approximately 5–6 patients per month to achieve 100 study participants within an 18-month time period. However, recruitment has been hampered due to the COVID pandemic, and therefore, we now expect recruitment to take approximately 24 months.

### Sample size {14}

We calculated our sample size for our primary outcome, the proportion of patients in GC-free remission. Based on two previous RCTs studying the efficacy of MTX with regard to GC-free remission [20, 21], we assumed conservative, but still a clinical important, GC-free remission proportions of 70% versus 40% at week 52 (MTX versus placebo respectively). To calculate sample size, STATA/IC version 13 for windows was used, a chi-square test with a power of 0.80, a two-tailed alpha of 0.05, a 1:1 allocation ratio, and correction for continuity. This resulted in a total sample size of 98 patients, 49 per treatment arm. We calculated the sample size for different GC-free proportions in both groups. As shown in Table 2, with a sample size of at least 98, we are on the safe side of finding a clinically relevant difference between groups, but also still on the feasible side. We increased the sample size to 100 as we expect a maximal drop-out of < 5%, as has been the experience in our center before [36].

**Table 2:** Patients needed for different effect sizes

**Proportion of GC-free remission**		**Placebo group**						
		0.5	0.45	0.4	0.35	0.3	0.25	0.2
**MTX group**	0.8	90	70	56	44	38	32	26
	0.75	132	96	72	56	46	38	32
	0.7	206	136	98	74	58	46	38
	0.65	366	212	140	98	74	56	44
	0.6	816	372	214	140	98	72	56
	0.55	3210	824	372	212	136	96	70
	0.5		3210	816	366	206	132	90

STATA/IC 13, a two-tailed α of 0.05, power of 0.80, correction for continuity

### Recruitment {15}

To stimulate patient enrolment, we placed information regarding the study on the website of the Dutch Arthritis Society (Dutch: ReumaNederland) and the Sint Maartenskliniek. Every year, the diagnosis PMR is made in approximately 100 patients in the Sint Maartenskliniek and around 50 patients a year fulfill the 2012 EULAR/ACR criteria. Because not every patient will want to, or is eligible to, participate in this trial, other centers, and general practitioners are encouraged to refer patients to the Sint Maartenskliniek. Furthermore, collaboration with other rheumatology clinics will be sought to enhance patient enrolment. In case of exclusion of subjects if another diagnosis appears to more likely, additional subjects will be recruited, to ensure a minimum of 100 evaluable patients in the analysis.

## Methods: assignment of interventions: allocation

### Sequence generation {16a}

The treatment allocation sequence will be generated by computer-generated random numbers and we will stratify in 4 groups based on sex and elevation of APR (either an ESR ≥ 70 mm/h or a CRP ≥ 25 mg/L). Previous studies show that sex and serum level of inflammatory parameters before treatment may be predictors of PMR relapse during the first year of treatment; thus, we wanted to make sure these variables were balanced between the MTX and placebo group [3, 25]. A variable block size will be used to reduce predictability of the randomization sequence, while maintaining balance in numbers. The details of this randomization sequence and treatment allocation will be unknown to all personnel part of the research team and only known to colleagues of the pharmacy.

### Concealment mechanism {16b}

MTX and placebo pills will be identical in packaging. Furthermore, the capsules will be identical to each other in appearance. Patients will receive the study medication from the pharmacy that is located at the Sint Maartenskliniek Nijmegen.

### Implementation {16c}

The treating physicians will enroll patients. The interventions will be assigned by the pharmacy that has the document with the allocation sequence.

## Methods: assignment of interventions: blinding

### Who will be blinded {17a}

Patients, researchers, and all health care providers (HCP), including nurses, and (research) physician(s) (assistants), will be blinded for 52 weeks.

### Procedure for unblinding if needed {17b}

If MTX is not tolerated during the trial period of 4 weeks (as assessed by laboratory values and AE), the dosage is not raised further, or dose can be lowered without unblinding patients or caregivers. MTX capsules of 5 mg will be used to allow easier dose adjustment without unnecessarily unblinding participants or HCP. Unblinding is only done on request of a treating physician when this is needed for adequate treatment. This request will be made to the pharmacist and subsequent unblinding will be done by the pharmacist.

## Methods: data collection and management

### Plans for assessment and collection of outcomes {18a}

See Table 3 for all the assessments and collection of outcomes that will take place during this study. At baseline, patient characteristics and physical examination will be performed. Patient characteristics include age, gender, smoking habits, alcohol use, and previous medical history. Disease characteristics that will be assessed include PMR-specific symptoms and regions, pre-treatment symptoms duration, involvement of systemic symptoms, and treatment prior to inclusion. Physical examination will include at least length, weight, blood pressure, pulse rate, and temperature.

**Table 3:** Schedule of enrolment, interventions, and assessments

Timepoint	Enrolment	Post-allocation (weeks)								Close-out
	***-t***_***1***_	***Baseline***	***4***	***8***	***12***	***16***	***24***	***32***	***42***	***52***
**Enrolment**										
**Eligibility screen**	X									
**Informed consent**	X									
**Allocation**		X								
**Intervention**										
***Methotrexate***										
***Placebo***										
**Assessments**										
**Demographics, medical history, medication overview, RF, ACPA, AP**		X								
**Disease characteristics, physical examination, PMR-AS, AE monitoring, PROs**^a^		X	X		X		X	X		X
**CRP, ESR, total blood count, creatinine, ALAT, serum for storage**		X	X	X	X	X	X	X	X	X
**GTI, including serum glucose, HbA1c, LDL**		X			X		X			X

*Abbreviations: RF*, rheumatoid factor; *ACPA*, anti-cyclic citrullinated peptide; *AP*, alkaline phosphatase; *PMR-AS*, polymyalgia rheumatica disease activity score; *AE*, adverse events; *PROs*, patient-reported outcomes; *GTI*, Glucocorticoid Toxicity Index; *HbA1c*, glycated hemoglobin; *LDL*, low-density lipoprotein; *CRP*, C-reactive protein; *ESR*, erythrocyte sedimentation rate; *ALAT*, alanine aminotransferase

^a^Transition, patient acceptable symptom state, EQ-5D-5L, HAQ, and PROMIS-PF

At baseline and every follow-up visit, the PMR-AS will be assessed. To date, there is no consensus-based measure for disease activity in PMR, although the PMR-AS seems most evidence based [38]. Previous research showed the PMR-AS may discriminate remission from relapse in clinical practice if a cut-off of 10 is used [32–35]. The PMR-AS is calculated from CRP measurements (mg/dl), the duration of morning stiffness (MST, minutes), the ability to raise the arms ((elevation upper limb; EUL); 3 to 0: 3 = no elevation possible; 2 = elevation possible below shoulder girdle; 1 = up to shoulder girdle; 0 = full elevation possible), physician’s global assessment (physician’s visual analogue scale (VAS ph); 0 to 10), and the patients’ assessment of pain (patient’s visual analog scale (VAS p); 0 to 10). The total score will be calculated with the formula as described by Leeb et al. [34]. Primary response will be defined as ≥ 70% improvement from baseline in PMR Visual Analogue Scale and duration of morning stiffness, combined with normal CRP or ESR. Remission during the visits will be defined as a PMR-AS < 10 [32–35]. Relapse will be defined as judged by the treating physician. AE will be assessed at every visit. Additionally, GC-related AE are assessed by using a modified version GTI as discussed under SPIRIT header {12} [37].

#### COVID study visits

Due to the COVID pandemic, study visits will be done without physical appointments when possible. Study visits at weeks 0, 32, and 52 will be done physically, as these are necessary to accurately assess and analyze primary and secondary outcomes. Other study visits will be performed by telephone and laboratory assessment may be performed at local laboratories, based on physician and patient shared decision making. We think this is possible since the (main) physical examination, the EUL, and other parameters (e.g., pain and morning stiffness) may be done by telephone.

### Plans to promote participant retention and complete follow-up {18b}

Contacting the research physician will be made very accessible for patients. Patients will be seen as soon as possible when they experience a relapse. Furthermore, it will be made clear that prednisolone dose can (quickly) be raised in case of a relapse. The reason for withdrawal will be asked but patients do not need to provide the answer if they prefer not to disclose their reason.

### Data management {19}

A CASTOR EDC database will be used to store all study data anonymously. CASTOR also enables an audit trail. Data entry will be done by a research assistant. Data will be checked by double entry in 10% of patients. Before analysis, data will be checked on completeness and range checks will be made to detect any (potential) outliers. Data will be stored for 25 years after the end of the study.

### Confidentiality {27}

All data will be collected and stored anonymously in a CASTOR database. Data will be coded and kept based on the rules for good clinical practice (GCP) and Dutch law. Only the trial researchers will have access to the CASTOR database through a personal password.

### Plans for collection, laboratory evaluation, and storage of biological specimens for genetic or molecular analysis in this trial/future use {33}

Study data will be stored for 25 years after the end of the study period. Blood samples for this study will be stored for the duration of this study period to do additional testing if necessary. Additional permission will be asked to use data and additional blood samples for future research in the field of PMR, as described in the patient information brochure. Blood samples for future research will be stored for 10 years. Blood samples will be sent to the laboratory of the SMK and anonymously coded and delivered. The database managers keep a unique code list at a secured location which is only accessible to the database manager and the principal investigator. Additional research on material will only take place after medical ethical approval.

## Statistical methods

### Statistical methods for primary and secondary outcomes {20a}

All statistical analyses will be performed using STATA/IC version 13 for Windows. Results will be analyzed on an intention-to-treat approach, and an additional per protocol sensitivity analysis will be done as described under SPIRIT header {20c}. Descriptive statistics will be provided using mean and standard deviation (SD), median and interquartile range (IQR) or frequencies / percentages as appropriate.

The primary outcome, the proportion of patients in GC-free remission after a total of 52 weeks, will be compared using a Cochran–Mantel–Haenszel procedure with stratification for sex and APR as discussed under SPIRIT header {16a}.

Of the secondary outcomes, the proportion of patients in GC-free remission at week 32, the proportion of patients with low dose GC ≤ 5 mg at week 32 and 52, proportion of the patients that have a relapse during follow-up, and proportion of patients that had MTX/placebo dose adjustment at week 52 will be analyzed in the same manner as the primary outcome. Time to remission and time to first relapse will be compared using Kaplan-Meier analysis. GC cumulative dose will be compared using an independent *t*-test or Mann-Whitney test. Number of relapses will be compared using Poisson regression. Change in ESR and CRP, PMR-AS, transition and PASS, VAS, EQ-5D, HAQ, and PROMIS-PF will be compared using an independent *t*-test or Mann-Whitney test; Glucocorticoid Toxicity Index will be compared using an independent *t*-test or Mann-Whitney test; number of AE will be compared using an independent *t*-test or Mann-Whitney test. A *p*-value < 0.05 will be considered significant.

### Interim analyses {21b}

No preplanned interim analyses will take place. On request of the DSMB, an interim analysis can be performed for safety reasons.

### Methods for additional analyses (e.g., subgroup analyses) {20b}

We will stepwise study the treatment-modifying effect of covariates and the primary (and secondary) study parameter(s) by starting with models with the (various) dependent outcome variables and independent treatment variable, and thereafter adding and removing covariates one at a time. We will first study the correlation with the stratification factors: sex and ESR and CRP level before treatment as covariates. Thereafter, we will use the stratification of sex and pre-treatment CRP/ESR in a model and stepwise add and remove age, smoking, alcohol use, BMI, pre-treatment symptom duration, and time to initial response as covariates.

Economic evaluation will be performed in a secondary cost-utility study guided by national recommendations [39]. Costs will be determined by multiplying units of medication and rheumatology appointments by costs per unit. QALY will be calculated using an area under the curve (AUC) method using utility scores converted from the EQ-5D-5L [40, 41].

### Methods in analysis to handle protocol non-adherence and any statistical methods to handle missing data {20c}

Patients in which an alternative diagnosis is considered more likely, e.g., due to lack of response to prednisolone (as discussed under SPIRIT header {11a}), are excluded from the primary analyses. After this exclusion, patient data will primarily be analyzed in an intention-to-treat (ITT) manner. Efficacy-related outcomes will also be analyzed in a per-protocol (PP) manner. Patients will be excluded from the PP analysis if they either deviated from the tapering protocol more than eight weeks, are treated with prednisolone ≥ 20 mg/day for 2 weeks due to other complaints than PMR (after initial response to prednisolone), or are allocated to MTX group and were not treated with at least MTX 15 mg/week for at least 6 months. Potential reasons for drop-out will be noted and assessed with regard to the rise of attrition bias due to loss to follow-up.

The number of missing values and the role of the corresponding variables will be assessed. Missing value patterns will be analyzed using visualization (e.g., histograms), potential causes for missing (e.g., treating physician and PP adjustments) will be examined, and supportive testing using Little’s test for Missing Completely At Random will be used [42]. If missing values are limited in number and importance (of the corresponding variable), a complete case analyses, per analysis per outcome, can be considered. If missing values occur more often, or the corresponding variable is deemed important, and we assume data to be Missing (Completely) At Random, then an imputation technique will be considered. Imputation will be considered separately for ITT and PP analyses. The imputation technique used will depend on the missing values, with a preference for multiple imputation (using chained equation) as opposed to single imputation, like Last Observation Carried Forward [43].

### Plans to give access to the full protocol, participant-level data, and statistical code {31c}

Full public access to the full protocol will be granted. After the initial analyses of study data, access to anonymized participant-level dataset and statistical code may be granted upon request.

## Oversight and monitoring

### Composition of the coordinating center and trial steering committee {5d}

The coordinating center is the Sint Maartenskliniek: principal investigator is Dr. Aatke van der Maas, rheumatologist-epidemiologist, and coordinating investigator is Thomas Bolhuis, MD.

### Composition of the data monitoring committee, its role, and reporting structure {21a}

Stringent monitoring is not formally necessary as this study is classified as negligible/low risk. Nevertheless, an independent Data Safety Monitoring Board (DSMB) will be installed to monitor the inclusion progress of the study every 6 months. Members of the DSMB are independent from the study and include a pharmacist, an internist, a rheumatologist, and a methodologist. Their role is to monitor the feasibility and safety of the study, e.g., inclusion rate and the occurrence of (serious) adverse events((S)AE). Meetings will be planned every 6 months from the time of first inclusion. The principal and coordinating investigators will be present during meetings.

### Adverse event reporting and harms {22}

All (S)AE reported spontaneously by the subject or observed by the investigator will be recorded. The (S)AE will be reported to the medical ethical authorities according to Dutch legislations. An annual safety report will be written and submitted to the competent authority throughout the duration of the clinical trial. Furthermore, AE will be discussed in the DSMB (as discussed under SPIRIT header {21a}) and checked by the monitor (as discussed under SPIRIT header {23}).

### Frequency and plans for auditing trial conduct {23}

As this is a low risk study, monitoring of the trial will be performed once per year. The assigned monitor is independent from the study and “BROK” certified conform Dutch guidelines. Monitoring will consist of checking rate of inclusion, drop-out, the investigator site file, informed consent for 25% of participants, in- and exclusion criteria for 10% of participants, source data of 1–10% of participants, SAE in 1% of participants, and verify SAE in 10% of SAE. The monitor will write a report, which will be assessed and signed by the principal investigator.

### Plans for communicating important protocol amendments to relevant parties (e.g., trial participants, ethical committees) {25}

Any protocol amendments will first be communicated to the medical ethical committee. After approval by the medical ethical committee, the protocol amendments will be communicated to the study participants if the amendments apply to the patients. Depending on the degree of amendments, they will also be communicated to all other relevant parties such as the treating physicians, research personnel, trial registries, and regulators.

On 05-07-2021, the trial was amended on the following points: addition of the outcomes low-dose GC remission and direct healthcare costs, visits (except for week 32 and 52) may be performed by telephone (due to COVID), intention-to-treat, per-protocol, and missing data analysis, a secondary cost-utility analysis, and removal of the baseline chest X-ray. At this moment, 31 patients were included in the trial, five had finished the trial, and one was unblinded due to a serious AE. On 20-07-2021, the trial was amended to include Gelre Ziekenhuizen, making the study a multicenter RCT.

### Dissemination plans {31a}

The main findings of clinical trials will be authored by investigators of the Department of Rheumatology from the Sint Maartenskliniek and submitted for publication in a peer-reviewed journal within 12 months of study completion. Researchers who have made significant contributions to the study will be included in the list of authors. In addition, key outcomes are to be made publicly available within 12 months of study completion by posting to the results section of the primary clinical trial registry. Furthermore, a layman’s summary of the results will be posted on a free-to-access, publicly available, searchable institutional website of the Sint Maartenskliniek and will also be disseminated to all patients who participated in the study.

## Discussion

With this double-blinded placebo controlled superiority design, our aim is to investigate whether MTX 25 mg/week is efficacious and leads to disease remission and GC-sparing in PMR. Several choices for the design are motivated above, but we would like to discuss some aspects and challenges of the study design in more detail below.

First, the selection of patients: in an ideal setting we would like to include all patients with clinical PMR in the study. However, a clinical diagnosis of PMR may be less specific for PMR than the EULAR/ACR classification criteria and there may be much heterogeneity among rheumatologist regarding clinical diagnosis. Thus, using these criteria will—although generalizability and inclusion will be somewhat hampered—improve homogeneity, both within the study, and when comparing with other studies. Of note, these criteria have a moderate sensitivity and specificity for PMR [2]. Therefore, not all patients with clinically diagnosed PMR can be included, e.g., patients with normal acute phase parameters. However, these patients may represent a subset of PMR patients with a different (more benign) disease course, possibly benefitting less from add-on MTX [44]. In- and exclusion criteria for this trial are less strict when compared to other PMR trials in the Netherlands (a Leflunomide trial (clinical trial identifier: NCT03576794) and a sarilumab trial (clinical trial identifier: NCT03600818)). Firstly, newly diagnosed PMR for this trial is defined as GC use ≤ 8 weeks, which we assume more feasible and reflecting the true newly diagnosed PMR patients we see in daily clinical practice, as opposed to a shorter treatment duration. Secondly, exclusion based on conditions interfering with pain and movement evaluation is left to treating rheumatologists, for example fibromyalgia is not a formal contra-indication, unless this might interfere with assessments. We assume these exclusion criteria improve feasibility and generalizability. Lastly, eligibility for treatment with MTX is left to judgment of the treating rheumatologist/research physician. This was decided because there is a lot of experience with MTX in rheumatology practice, and this choice is supported by the Dutch guidelines for MTX treatment which does not formulate absolute contra-indications [31].

We have considered also including PMR patients relapsing during tapering of GC. Indeed, to date, even though MTX is recommended for patients at risk for worse prognosis in guidelines for management of PMR, the evidence of a GC-sparing effect of concomitant MTX in PMR patients who relapse is not strong [7]. Furthermore, including PMR patients who relapse in our study would have the advantage of a higher inclusion rate. However, in this study, we decided not to include PMR patients who relapse for several reasons. Firstly, it is uncertain if MTX efficacy will be the same in both these subgroups since effect modification may occur. Secondly, it is unknown what the optimal GC-dose cut-off point is at moment of relapse for starting concomitant MTX, since at lower GC doses the possible benefits of MTX may not outweigh the risks. Therefore, we chose to include recently diagnosed PMR patients only.

Another point we would like to address is the primary outcome GC-free remission, considering our GC tapering based strategy study. We deliberately chose a composite endpoint of disease activity (remission defined as PMR-AS< 10) and treatment (GC-free), as we think this is in more meaningful than an outcome based on disease activity or treatment alone. Remission defined as a PMR-AS< 10 is both a subjective and objective outcome and includes both patients’ and physicians’ view of PMR disease activity. In our study if a patient experiences a relapse, this will be ameliorated by increasing prednisolone to the pre-relapse dose. This may lead to second order effects such as increased cumulative GC dose or being symptom free but not GC free. Therefore, incorporating both disease activity and current GC treatment may counteract a second order effects as well.

Concerning the intervention, the choice for MTX capsules instead of injections has already been motivated under SPIRIT header {6b}, the accelerated GC-tapering on the other hand will be discussed further here. In this study, we chose an accelerated GC-tapering regime of 24 weeks, which is approximately twice as fast as usual care. Due to this increased tapering speed, and minimal GC treatment, we will be more likely to find a treatment effect of MTX if it exists when compared to regular care tapering. Furthermore, two previous RCTs examined MTX using a comparable tapering scheme; therefore, our results may be more easily compared with those [22, 26]. In addition, increased tapering may lead to better insight in the ideal length or speed of a GC tapering scheme for PMR. One aspect we did not discuss before is the possible increased chance of relapsing during tapering. In discussions with patients in the design phase of our study, patients were willing to accept this risk if contact with the physician is easily accessible and prednisolone may promptly be raised to the pre-relapse dose. Furthermore, since a diagnosis has already been made, both the patient and research physician may be able to identify a relapses quicker.

A final challenge of this study is the COVID-19 pandemic as both inclusion and follow-up of patients are hampered by distancing measures. To guarantee patient safety and reduce risk of COVID19 infection and spreading, we chose to perform study visits digitally where possible and needed. Despite limited physical appointments, we do think that adequate study assessments will be made as most assessments can be collected reliably by telephone and the physical visits required are limited.

In conclusion, a positive outcome of this trial will have significant implications for the management of PMR. If this trial proves efficacy of MTX in PMR patients in an early disease phase, it will lead to improved treatment of PMR and provide a GC-sparing agent for all patients.

## Trial status

Protocol version 2.1, date 20-07-2021. Recruitment started at 02-03-2020 and is expected to be completed by 08-2022.

## Abbreviations

ACR: American College of Rheumatology
ACPA: Anti-citrullinated protein antibodies
AE: Adverse event
ALAT: Alanine-amino-transferase
CK: Creatinine kinase
CRP: C-reactive protein
DMARD: Disease-modifying anti-rheumatic drug
DSMB: Data Safety Monitoring Board
EMA: European Medicines Agency
EQ-5D: Europol Five Dimensions Questionnaire
ESR: Erythrocyte sediment rate
EudraCT: European Drug Regulatory Affairs Clinical Trials
EULAR: European League Against Rheumatism
GC: Glucocorticoids
GCA: Giant cell arteritis
GCP: Good Clinical Practice
GDPR: General Data Protection Regulation; in Dutch: *Algemene Verordening Gegevensbescherming* (AVG)
GTI: Glucocorticoid Toxicity Index
(M)HAQ: (Modified) Health assessment questionnaire
IC: Informed consent
LDH: Lactate dehydrogenase
METC: Medical Research Ethics Committee (MREC); in Dutch: *medisch-ethische toetsingscommissie* (METC)
MTX: Methotrexate
NFU: Netherlands Federation of University Medical Centres
OMERACT: Outcome Measures in Rheumatology (workgroup)
PASS: Patient acceptable symptom state
PMR: Polymyalgia rheumatica
PMR-AS: Polymyalgia rheumatica activity score (measure for PMR-specific disease activity)
PRO: Patient-reported outcome
PROMIS-PF: Patient-Reported Outcome Measurement Information System Physical Function
QALY: Quality-adjusted life year
RA: Rheumatoid arthritis
RCT: Randomized controlled trial
RF: Rheumatoid factor
(S)AE: (Serious) adverse event
SUSAR: Suspected unexpected serious adverse reaction
UAVG: Dutch Act on Implementation of the General Data Protection Regulation; in Dutch: *Uitvoeringswet AVG*
VAS: Visual analogue scale
WMO: Medical Research Involving Human Subjects Act; in Dutch: *Wet Medisch-wetenschappelijk Onderzoek met Mensen*
